# DIONYSUS: a database of protein–carbohydrate interfaces

**DOI:** 10.1093/nar/gkae890

**Published:** 2024-10-22

**Authors:** Aria Gheeraert, Thomas Bailly, Yani Ren, Ali Hamraoui, Julie Te, Yann Vander Meersche, Gabriel Cretin, Ravy Leon Foun Lin, Jean-Christophe Gelly, Serge Pérez, Frédéric Guyon, Tatiana Galochkina

**Affiliations:** Université Paris Cité and Université des Antilles and Université de la Réunion, INSERM, BIGR, DSIMB, F-75015 Paris, France; Université Paris Cité and Université des Antilles and Université de la Réunion, INSERM, BIGR, DSIMB, F-75015 Paris, France; Université Paris Cité and Université des Antilles and Université de la Réunion, INSERM, BIGR, DSIMB, F-75015 Paris, France; Université Paris-Saclay, INRAE, MetaGenoPolis, 78350 Jouy-en-Josas, France; Université Paris Cité and Université des Antilles and Université de la Réunion, INSERM, BIGR, DSIMB, F-75015 Paris, France; Institut de biologie de l’Ecole normale supérieure (IBENS), Ecole normale supérieure, CNRS, INSERM, PSL Universite Paris, 75005 Paris, France; Université Paris Cité and Université des Antilles and Université de la Réunion, INSERM, BIGR, DSIMB, F-75015 Paris, France; Université Paris Cité and Université des Antilles and Université de la Réunion, INSERM, BIGR, DSIMB, F-75015 Paris, France; Université Paris Cité and Université des Antilles and Université de la Réunion, INSERM, BIGR, DSIMB, F-75015 Paris, France; Université Paris Cité and Université des Antilles and Université de la Réunion, INSERM, BIGR, DSIMB, F-75015 Paris, France; Université Paris Cité and Université des Antilles and Université de la Réunion, INSERM, BIGR, DSIMB, F-75015 Paris, France; Centre de Recherches sur les Macromolécules Végétales, University Grenoble Alpes, CNRS, UPR, 5301 Grenoble, France; Université Paris Cité and Université des Antilles and Université de la Réunion, INSERM, BIGR, DSIMB, F-75015 Paris, France; Université Paris Cité and Université des Antilles and Université de la Réunion, INSERM, BIGR, DSIMB, F-75015 Paris, France

## Abstract

Protein-carbohydrate interactions govern a wide variety of biological processes and play an essential role in the development of different diseases. Here, we present DIONYSUS, the first database of protein-carbohydrate interfaces annotated according to structural, chemical and functional properties of both proteins and carbohydrates. We provide exhaustive information on the nature of interactions, binding site composition, biological function and specific additional information retrieved from existing databases. The user can easily search the database using protein sequence and structure information or by carbohydrate binding site properties. Moreover, for a given interaction site, the user can perform its comparison with a representative subset of non-covalent protein-carbohydrate interactions to retrieve information on its potential function or specificity. Therefore, DIONYSUS is a source of valuable information both for a deeper understanding of general protein-carbohydrate interaction patterns, for annotation of the previously unannotated proteins and for such applications as carbohydrate-based drug design. DIONYSUS is freely available at www.dsimb.inserm.fr/DIONYSUS/.

## Graphical Abstract

**Graphical Abstract:**
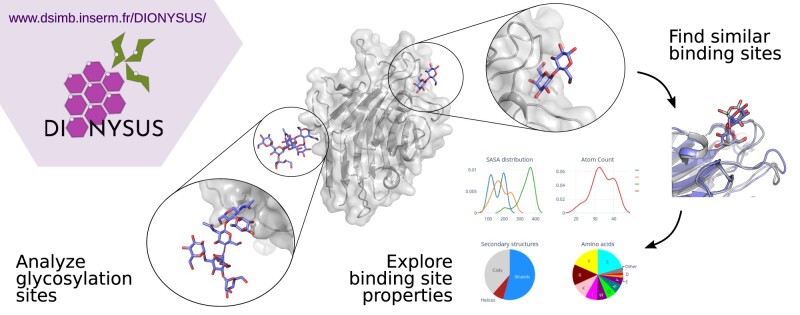

## Introduction

Carbohydrates are ubiquitous in enzymatic pathways and are the primary energy source in living organisms (1). Moreover, carbohydrates are abundantly present on the surface of living cells and thus are involved in cellular recognition and signaling (2–6). As a result, protein–carbohydrate interactions are related to various diseases, such as cancer tumor growth (7), and mediate a number of host–pathogen infections (8–11). Therefore, both carbohydrates and carbohydrate binding proteins are important targets for drug and protein design (12,13).

Despite the significant contribution of the glycoscience community to the annotation of carbohydrates and carbohydrate binding proteins (14–17), the comparison of protein-carbohydrate interfaces remains a complex task mostly due to the chemical diversity of carbohydrates, the flexibility of protein-carbohydrate interfaces and the experimental challenges of their resolution (18,19). The first database providing information on the resolved structures of protein-carbohydrate complexes, ProCarbDB (17), was released in 2021 but is no longer available at the time of publication of this article. Information on carbohydrate binding sites (CBS) can also be implicitly retrieved from other non-specific databases on protein-ligand interactions such as BioLiP (20,21) or Binding-MOAD (22). Still, they often miss specific carbohydrate properties and do not distinguish between covalent and non-covalent interactions. Therefore, our primary goal in this study was an extensive systematic annotation and classification of all experimentally resolved protein–carbohydrate interfaces.

We have retrieved all the carbohydrate-containing entries available in the Protein Data Bank (23) and provided an exhaustive annotation of protein, carbohydrate and interaction site properties according to specialized databases and information extracted from 3D structure. Then, we performed a pairwise comparison and clustering of carbohydrate binding sites according to their geometry for a selected high quality subset of non-covalent protein-carbohydrate interfaces. DIONYSUS allows the user to efficiently perform comparative analysis of different sugar binding sites, to explore carbohydrate specificity for various protein types, as well as to evaluate complex composition and resolution quality through a user-friendly interface.

## Materials and methods

### Data extraction

*Identification of carbohydrate-bringing compounds*. We have extracted all the sugar-like compounds present in the Chemical Component Dictionary of the wwPDB (23). Our final list includes >3k different components, including nucleosides and their derivatives. Importantly, we have explicitly excluded RNA and DNA-forming polymer components from this study and invite users to consult specialized databases such as DNAproDB (24) for the analysis of corresponding molecular interfaces. Among identified compounds, 168 molecules with exhaustive carbohydrate-specific annotation in the PDB (25) were denoted as ‘core dataset’ (see Supplementary Data and Supplementary Figure S4 for the details).

*Retrieval of general information*. We used an API to download all mmCIF structure files with residue-level annotations containing at least one protein chain and one of the identified saccharides from the EMBL-EBI server (26). Each protein structure file was parsed using the GEMMI module (26). For structures containing several models and alternate locations, we treated each combination of a model and an alternate location individually. We report essential information such as PDB code, method of resolution, comments about the quality of the resolved structure, UniProt ID, organism, functional information, complete sequence, missing residues and missing atoms. For the web interface, we used the RCSB Saguaro Web Application (27) to recover protein information including secondary structure, missing residues, artifacts, mutations, metal coordination, hydropathy, disorder, and different domain annotations if they exist.

*Interface analysis*. For each carbohydrate residue, we analyzed its geometry and physico-chemical properties of the protein surface region in its proximity. Since protein–carbohydrate interactions are often flexible and contain resolution errors, we adopt a purely geometric approximation of a carbohydrate binding site for non-covalent protein-carbohydrate interactions, similar to that used in works on peptide–protein and drug–protein interfaces (28). For a given carbohydrate moiety (monomer or part of a more complex ligand), we consider its binding site to be formed by the heavy atoms located on the protein surface (solvent accessible surface area (SASA) >0 according to freeSASA (29)) and closer than 7 Å to any atom of the carbohydrate ring. According to this definition, if a complex glycoside interacts with a protein only through its aglycon part, it would not be classified as forming a protein-carbohydrate interface. In case of alternate locations of protein or carbohydrate residues, we generate distinct binding sites for each combination of model and alternate location. For each binding site we report chains involved in binding and cross-reference our binding site database with BioLiP2 (20,21), CAZy (30) (carbohydrate-active enzymes), UniLectin (14,31), SAbDab (32), LectomeXplore (33), Uniprot (34), GAG-DB (35). Finally, we use the same geometrical approximation to analyze protein surface region in the proximity of carbohydrates covalently attached to protein residues.

### Annotation of protein–carbohydrate interfaces

PDB contains protein–carbohydrate complexes of different nature, including non-covalent ligand binding, glycosylated proteins and glycopeptides, and reaction intermediates in sugar enzymes. We attribute each carbohydrate residue to one of the three types according to their interaction mode: free carbohydrates (involving strictly non-covalent interactions), glycosylation heads (representing a sugar moiety covalently attached to a protein residue), and glycosylation bodies (referring to a carbohydrate portion covalently linked to the glycosylation head, potentially engaging in non-covalent interactions with protein residues). We use the same approximation for other covalent interactions, such as covalently linked aglycons (see Supplementary Data for the details).

We use both author-provided annotations and our computational assessments to validate the presence of covalent bonds between carbohydrates and proteins. The author's annotations can reveal inaccurately resolved glycosylations (e.g. 7QTV, where the N-glycosylation between N205 chain B and NAG401 chain B exhibits a C-N bond length of 3.4 Å). At the same time, sometimes they do not correspond well to the structural evidence (e.g. in structure 6S08, an O-glycosylation between T272, chain A, and FUC1 chain C was not reported, but distance calculation suggests its presence). For each bond, we assess its potential covalency by considering the sum of the upper bounds of experimental covalent radii (36) for the atoms involved, allowing for some margin (1.1 times the sum of upper bounds). Any structure containing a mismatch between the two methods is annotated accordingly. Additionally, we detect clashes between two atoms when their distance is less than half the sum of their experimental covalent radii, and we parse close contacts using the *_pdbx_validation_close_contact* section of the mmCIF file.

For glycosylation sites, we consider four categories: N-glycosylations involving a C-N bond between the carbohydrate and an asparagine or arginine residue, O-glycosylations defined as a C-O bond between the carbohydrate and either a threonine, serine, tyrosine, hydroxylysine or hydroxyproline, and C-glycosylations characterized by a C-C bond between the carbohydrate (typically mannose) and a tryptophan residue. Any other covalent bond between carbohydrates and proteins not fitting these categories is assigned to a separate class: undetermined. This category includes artifacts in PDB structures, covalent intermediates in sugar enzymes, covalently linked aglycons, or S-glycosylations (see section ‘Difficult cases in annotation of glycosylation sites’ of Supplementary Data, Supplementary Figures S1–S3).

For each CBS, we report its size in terms of atom number and solvent accessible surface area. Additionally, we provide information about the presence of other carbohydrates, nucleic acids or small molecules within the 7 Å cut-off, along with details regarding the chains and amino acids to which the carbohydrate binds. Carbohydrate binding sites often involve multiple protein chains (in particular, in antibodies and toxins). We consider all the chains involved in the interface formation and annotate the corresponding CBS accordingly. We report binding site composition in terms of amino acid types and secondary structure (helices, strands, and coils). Finally, we evaluate reliability of the resolved structure in terms of missing atoms in the carbohydrate ring/complete structure and incomplete occupancy of the carbohydrate.

The subset of protein-carbohydrate interfaces of high quality is denoted as a ‘Refined dataset’ and comprises structures with no artifacts detected by our analysis, i.e. satisfying the following conditions:

- The structure resolution is better than 3 Å for X-ray and EM structures
- No resolution problems are detected in the binding site proximity (no clashes/close contacts between any ligand atom and protein)
- There are no missing atoms in the protein binding site or in the carbohydrate compound
- At least 20 protein atoms participate in the binding site formation according to our definition
- The corresponding binding site is reported as biologically relevant according to BioLiP2

Such filters allow us to exclude from consideration a majority of crystallographic adjuvants, even though the automated protocol of BioLip2 can potentially miss out some binding sites of biological interest.

Information on carbohydrate and protein content is available at the ‘About’ page, which is updated dynamically.

### Mapping to other databases

All the extracted PDB chains were mapped to UniProt (34), allowing us to obtain protein IDs in various databases including Gene Ontology (37), Enzyme Commission number in EXPASY (38), BRENDA (39) and information on the location of active sites and glycosylation sites and alternative PDB structures of the same protein. We have also retrieved residue-level annotations of protein domains according to Pfam (40), SCOP2 (41), CATH (42) and ECOD (43,44). Additionally, each entry was cross-referenced with several carbohydrate-specific databases such as UniLectin3D (14) for lectins, LectomeXplore (33) for predicted lectins, CAZy (30,45) for carbohydrate-active enzymes, SAbDab (32) for antibodies, GlyGen (46) and GlyConnect (47). Furthermore, we linked the binding site database to BioLiP (20,21), which additionally provides binding affinities for certain CBS through external databases like Binding MOAD (22), PDBBind (48) and BindingDB (49).

Finally, leveraging the resulting cross-annotations, we define four distinct categories of binding sites present in DIONYSUS (Supplementary Figure S3). First, we identify lectin binding sites as those situated within protein chains classified as lectins by Unilectin3D, whilst removing active sites based on Uniprot annotations. Then, we define enzyme active binding sites, as those located in structures reported in CAZy and aligned with active sites according to UniProt annotations. We distinguish different CAZy classes: Glycosyl Hydrolases, Glycosyl Transferases, Polysaccharide Lyases, Carbohydrate Esterases and Auxiliary Activities. Carbohydrate Binding Modules (CBMs) attached to enzymes and responsible for non-catalytic carbohydrate recognition are treated separately. Finally, we define antibody binding sites as those cross-referenced within a protein, denoted as heavy or light antibody chain in SAbDab. All the remaining binding sites are categorized as ‘Others’.

### Binding site alignment and similarity score

To explore structural similarities among CBS, we used a modification of the non-sequential structural alignment algorithm previously developed for off-target drug binding detection (50,51) and successfully applied to screening of protein-peptide interactions (28). The details of the method implementation are provided in Supplementary Data and in the ‘About’ section of the website. The code is available at: https://github.com/DSIMB/CompareCBS.

### Redundancy elimination

Protein-ligand complexes are frequently crystallized as oligomers, and the exact same protein-ligand complex may have been resolved multiple times in the same pose. Still, the same protein chain can form multiple distinct binding sites for the same carbohydrate. We identified two CBS as identical if (i) the corresponding protein sequences share > 95% similarity, (ii) the interacting carbohydrate residue is the same and (iii) the non-sequential geometrical comparison of binding sites shows a score above 0.7 and coverage above 0.8 (see Supplementary Figure S6). Protein sequence identity clusters were retrieved from the PDB webserver (25) which uses the MMseqs2 (52) clustering algorithm. Recognition of identical oligosaccharides with different IUPAC names and subsequent glycan drawings were performed using Glycowork (53). Finally, to select a non-redundant representative subset of CBS we constructed a network where an edge links nodes representing identical sites (Supplementary Figure S6). We proceeded by iteratively choosing the node with the highest degree to be the ‘representative’ of its adjacent nodes. Subsequently, we eliminate these selected nodes from the network, continuing the process until the graph is empty.

### Clustering of sugar binding sites

We performed clustering of protein-carbohydrate interfaces for protein complexes with the most common carbohydrates (see section ‘Identification of carbohydrate-like compounds’) and focused only on CBS of high quality (‘Refined dataset’ as defined above),

We then perform all-vs-all pairwise non-sequential alignments of the representative sites in each functional class, except for ‘others’ (Supplementary Table S1). Using the resulting similarity matrices, we performed a hierarchical variation of spectral clustering (54,55) which was demonstrated to be efficient in managing complex similarity matrices (56). Technical details of clustering implementation are provided in Supplementary Data section ‘Hierarchical spectral clustering of carbohydrate binding sites’ (Supplementary Figure S6 and Supplementary Table S2).

### Search by keyword, protein sequence and structure

We have implemented database search by sugar- or protein-related keywords, by protein sequence and by protein structure. Keyword search analyzes all the protein, carbohydrate and binding site properties obtained as described above. Sequence search is performed using the *ggsearch36* tool in the fasta package (57) and searches among all the protein chains found in contact with at least one sugar moiety. Structure search is implemented using kpax (58), which aligns a target protein against a subset of protein domains with unique ECOD IDs selected based on their involvement in protein-carbohydrate interaction and the best resolution among proteins with the same fold. To obtain an exhaustive list of protein structures of similar fold, the user is invited to use the best hit ECOD ID as a parameter for the advanced database search.

### Database interface and management

The DIONYSUS interface design is part of a standardized designed system developed by our team for our recently released web-servers and databases (www.dsimb.inserm.fr/pages/tools.html). DIONYSUS is developed using the Bootstrap framework and is fully responsive, ensuring an optimal user experience across various devices. DIONYSUS robustness was monkey tested using gremlins tool, which simulates random actions using JavaScript. Database creation is fully automated thanks to implementation of cron jobs and regular backups. Full database updates are programmed every several months, while the ‘News’ tab is constantly updated in sync with the PDB.

## Results

As of June 2024, DIONYSUS contains over 330k carbohydrate moieties in interaction with proteins representing more than 4k of distinct sugar-like molecules. These interactions are found in ∼50k experimental structures involving 22k different proteins according to UniProt ID. Among those, ∼102k are glycosylation sites while ∼159k represent free ligand binding.

### Web interface

DIONYSUS website provides five tools for database exploration, several examples of use in ‘Help’ page, methodological explanation and database statistics in ‘About’ and the used external databases in ‘Resources’. The tools allow to: (i) search the database by protein sequence, structure or using various annotations, (ii) compare a binding site to all representatives found in DIONYSUS, (iii) explore clusters of CBS and (iv) analyze glycosylation patterns of a protein in different structures as compared to UniProt annotations. Finally, we provide information on the constantly updating database content at the ‘News’ section of the home page. DIONYSUS database organization and main possible ways of its exploration are provided in ‘Help’ with shortcuts to main functionalities implemented in the main page.

### Search

**Figure 1.**
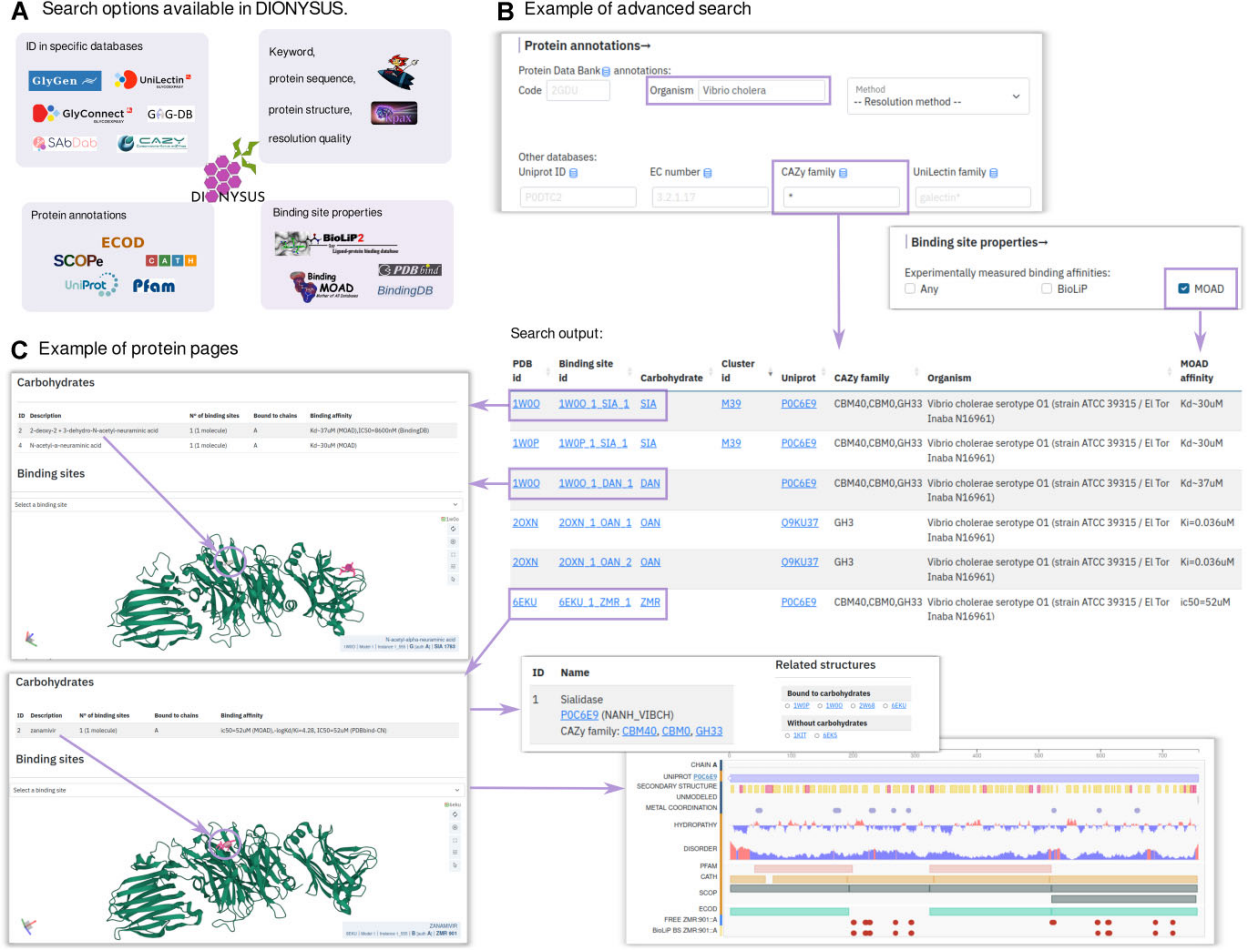
Different search options and examples of the search output. (**A**) Different databases linked to DIONYSUS and different types of database search implemented with logos of the corresponding engines (see Materials and methods for the details). (**B**) Example of the advanced search in DIONYSUS. Six binding sites were identified among all the protein-carbohydrate complexes expressed in *Vibrio cholera*, annotated in CAZy with available information on binding affinity in MOAD. (**C**) Protein pages of two sialidase complexes: with sialic acid (top, selected in pink), with Neu5Ac2en (top, pointed with arrow) and with a carbohydrate mimetic zanamivir (bottom, pointed with arrow). For PDB ID 6EKU we also provide additional information available on the protein pages such as identifiers in different databases, other structures of proteins sharing UniProt ID as well as sequence view with information on protein domain repartition and binding site location according to DIONYSUS and to BioLip.

The user can both perform a quick search using a keyword or protein sequence/structure, and benefit from a more detailed advanced search (Figure 1). We provide four categories of parameters to search through the database: by protein, by binding site properties, by carbohydrate properties or according to data reliability. Protein properties include annotations extracted from the PDB and from the specialized databases as described in ‘Materials and Methods’ (Figure 1A). The binding site properties section provides search by physico-chemical properties, secondary structure content, presence (or absence) of other carbohydrates or ligands (e.g. ions) and availability of experimental CBS affinities. Moreover, the user can select a minimal number of protein atoms participating in the interface formation in order to filter out weak interactions. In the carbohydrate section, the user can query for specific carbohydrates, restraining the interface type (i.e. free ligand or glycosylation), and select only monosaccharides or carbohydrates with a specific chemical function. Interactive autocompletion is implemented in order to facilitate the choice of desired three-letter PDB code for a sugar ligand. Using ‘Additional options’, the user can make a more complex request by searching for PDB entries containing a desired combination of carbohydrate residues. The data reliability section allows the user to filter CBS based on different criteria such as missing atoms or mismatch between author and structural information on covalent bonds, as well as to select biologically relevant ligands according to BioLiP. In all text fields, the user can use the wildcard * for partial keywords. For example, a query for proteins from *Vibrio Cholerae* annotated in CAZy and having binding affinity information available in MOAD (Figure 1B) results in six different binding sites found in two different proteins (with distinct UniProt IDs) and crystallized in four different PDB entries. Interestingly, sialidase (UniProt ID: P0C6E9) was obtained in complexes with carbohydrates and carbohydrate mimetics bound to the catalytic site (Figure 1C, shown with an arrow) and/or to the lectin-like domain (Figure 1C top, highlighted in pink). Search results can be downloaded both in .csv, .pdb or .xls format as well as in the form of a .zip archive containing raw .mmCIF structures of the selected complexes.

Finally, the user can also eliminate redundancy in the search results according to different annotations, selecting for instance a single CBS per by UniProt ID or per DIONYSUS cluster. Moreover, the user can eliminate structures with several models, alternate locations or keep only structures with a single CBS.

### Protein page

We provide multiple annotations for each protein chain of the PDB structure (Figure 1C). If a PDB entry contains several models (e.g. coming from NMR), the user can select each model using a drop-down menu. When the corresponding chain is bound to a carbohydrate, the user can select it to investigate individual chain properties according to different databases and to DIONYSUS annotation of the carbohydrate binding residues for each binding or glycosylation site (Figure 1C, right panel).

In the binding site section, the user can locate each CBS (per moiety) and recover information on its cluster, alternate locations if any, number of atoms and their SASA. In the ‘Related structures’ section, users can explore other resolved structures, which contain at least one chain with the same UniProt ID as the chain of interest. Those are grouped into two categories: structures containing a protein-carbohydrate interface (thus, annotated in DIONYSUS) and structures with no protein-carbohydrate interface (with a link to the corresponding Protein Data Bank page).

### Compare tool

**Figure 2.**
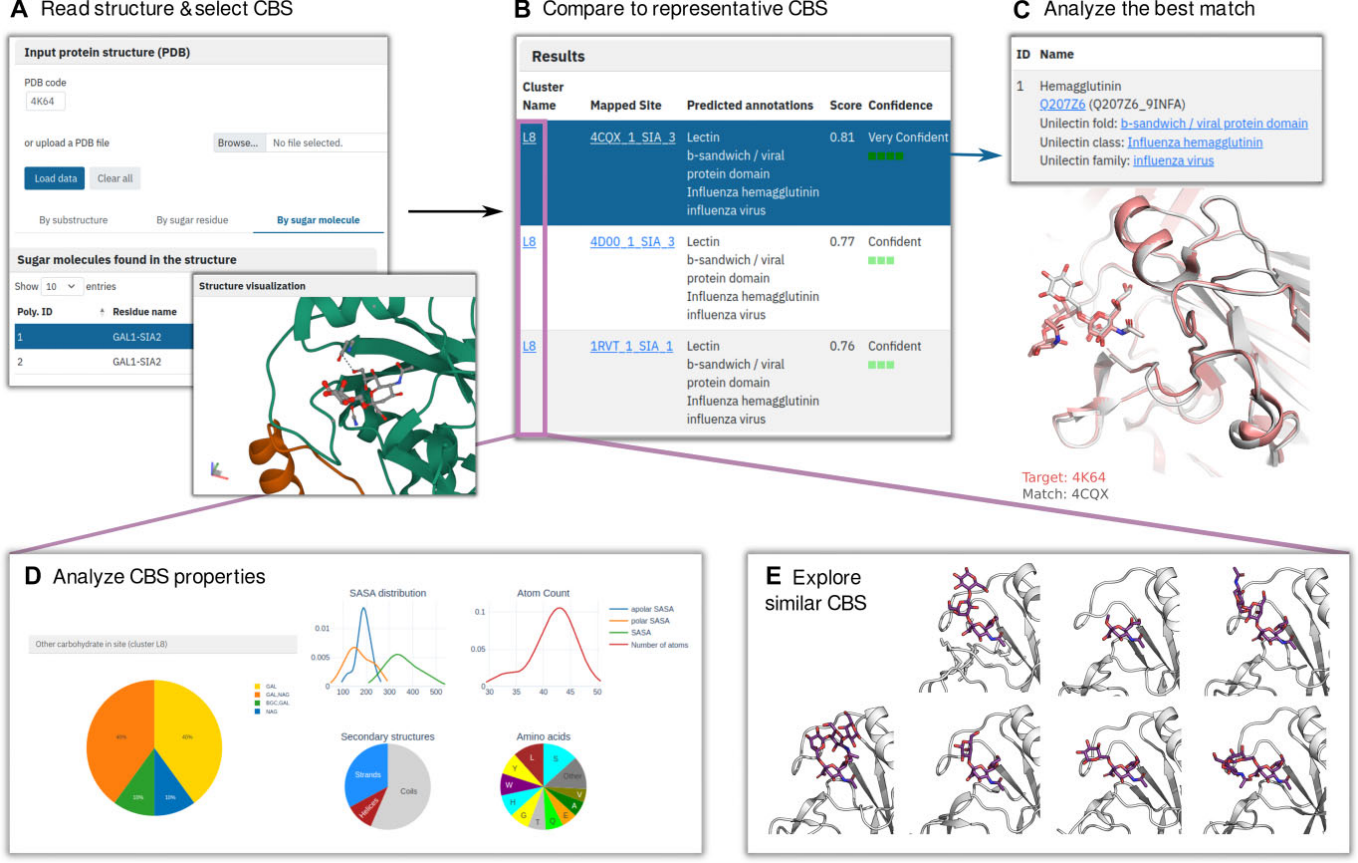
Example of comparison tool application to similar binding site search. (**A**) Compare tool identifies two carbohydrate molecules in the target structure (PDB ID: 4K64). We select the first one (shown in ‘Structure visualization’). (**B**) Results of comparison to representative CBS. The best match is obtained for the sialic acid binding site in the protein with PDB ID 4CQX. High scores are obtained for various proteins from cluster L8. (**C**) Information on the protein with the best match CBS and its superposition to the CBS in the target structure obtained using the downloadable PyMOL session. (**D**) Analysis of different properties of cluster L8. (**E**) Several other binding sites from cluster L8 in interaction with glycans of different composition. In accordance with panel D, most ligands bring galactose and/or *N*-acetyl-d-glucosamine residues in addition to sialic acid moiety.

A user can identify a cluster of similar protein-carbohydrate interfaces for a given PDB ID or for an uploaded PDB file. The submitted file is processed with an internal script and allows the user to select either a monosaccharide unit, or a complete carbohydrate ligand, as well as visualize the selection (Figure 2A). By clicking on ‘Compare’ button, the user can launch a two-step comparison process. First, we compare the CBS against all cluster representatives and select all clusters that scored higher than 0.5 (up to the top 5). Then, the target binding site is compared against every binding site from the selected clusters to refine our results. Finally, DIONYSUS ranks all the obtained scores and provides hyperlinks to the corresponding cluster pages for further exploration (Figure 2B) as well as a downloadable PyMOL session to investigate CBS superposition (Figure 2C, bottom). If a polysaccharide binding site is selected, the results are provided for each saccharide ring. For example, the best match for protein PDB ID 4K64 with no available annotation in UniLectin corresponds to sialic acid binding sites found in different hemagglutinin from Influenza (Figure 2C) suggesting its similar biological function.

### Explore clusters

In the ‘Clusters’ page, we provide a t-SNE 2D projection of all CBS clusters depicted by a symbol of the most prevalent carbohydrate according to the glycan nomenclature (59,60). The symbol size is logarithmically proportional to the number of elements within the cluster (Supplementary Table S2). The user can separately visualize proteins of different functional families, as well as select clusters of different quality based on the ratio between the intra-cluster and the inter-cluster scores (for the details, see Supplementary Data). By clicking on each symbol, the user can access the page dedicated to the corresponding cluster for further exploration. The user can further explore such characteristics of CBS as carbohydrate properties, binding site properties and data reliability within a specific cluster (Figure 2D) as well as compare them to the average properties of CBS in our database. The page header provides essential information, including a concise summary of the most prevalent carbohydrates, protein functions, ECOD topologies, CAZy families, and UniLectin families/kingdom/folds (if any CBS within the cluster has such annotations). Furthermore, the user can directly visualize a representative CBS and corresponding protein structure and download a PyMOL session to explore the resulting local superposition of CBS (Figure 2E).

### Glycosylation page

For a given UniProt ID, we provide all glycosylation patterns found in different resolved protein structures and in UniProt annotations. Glycosylations are depicted using the CPK color scheme with blue, red, and gray indicating N-linked, O-linked and C-linked glycans respectively. All other carbohydrate covalent binding sites are given in black. They correspond to either S-glycosylations, covalent intermediates or artifacts. The user can also hover over each dot to retrieve specific glycosylation annotations.

## Discussion

DIONYSUS is the first open-access database gathering exhaustive annotations of all the resolved protein-carbohydrate interfaces and providing information on their properties at different levels. Unlike existing databases that focus on proteins primarily recognized for their carbohydrate-binding functions, we adopt an agnostic view of protein-carbohydrate interactions. This perspective enables us to identify carbohydrate-binding proteins that may lack proper annotations in current databases (as illustrated in Figure 2), as well as carbohydrate-binding proteins not typically included in specialized datasets, such as transporters and porins involved in carbohydrate transport. Additionally, growing evidence suggests that sugars play a role in general protein-protein interactions. For example, sugars seem to promote the dimerization of FSH (61) and play a crucial role in stimulation of the spike protein RBD in SARS-CoV-2 (62). Therefore, the presence and organization of carbohydrate binding sites can be crucial even for the proteins normally classified as having different functions than carbohydrate binding. Providing such information also falls within the key scope of DIONYSUS.

During the database construction we have taken into account a range of features specific for protein-carbohydrate interaction and, therefore, not taken into account in general protein-ligand interaction analysis. First, we correctly treat sugar binding sites formed by multiple chains, and clearly distinguish covalent and non-covalent protein-carbohydrate interactions. Then, we are also the first to systematically report data on the presence of other molecule types in carbohydrate binding sites, which can be crucial for the existence and stability of such interactions (for instance, calcium ions). Finally, we account for the intrinsic flexibility of the protein-carbohydrate interfaces by explicit consideration and interactive treatment of the NMR structures.

We retrieved all the available annotations for proteins referenced in the specialized databases and provide tools facilitating attribution of new annotations. For a given carbohydrate-binding protein, DIONYSUS search and comparison tools allow gaining information on the nature and specificity of the corresponding interactions at different levels. First, using sequence information only, one can identify protein homologs found in interaction with a carbohydrate by running sequence-based search. Next, using an experimental protein structure, or a deep learning-generated model, one can use a structure-based search to identify sugar-bound proteins sharing similar folds. Finally, for proteins experimentally characterized in complex with a carbohydrate, we offer the possibility to compare corresponding binding sites with common interaction patterns in our database to potentially identify their specificity.

The development of DIONYSUS provides an important contribution to the ongoing big data and artificial intelligence revolution in glycosciences (63). In recent years, we have observed an increased interest of the bioinformatics community to the problem of carbohydrate binding site and glycosylation site prediction using state-of-the-art deep learning methods (64–66). These tools have great potential in aiding annotation and analysis of various carbohydrate binding proteins. However, their development and performance evaluation crucially depends on quantity and quality of the available data. We believe that redundancy analysis and extensive annotations provided in DIONYSUS will contribute to further improvement of the existing methods and will allow us to better assess their current limitations.

## Supplementary Material

gkae890_Supplemental_File

## Data Availability

DIONYSUS is freely available to any user via the following link: www.dsimb.inserm.fr/DIONYSUS, and does not require any login or registration.
