# Supplementary material for: DIONYSUS: a database of protein–carbohydrate interfaces
Source: Nucleic Acids Res. 2024 Oct 22;53(D1):D387–95. doi: 10.1093/nar/gkae890 (PMC11701518; doi:10.1093/nar/gkae890)
Supplement: gkae890_Supplemental_File [file gkae890_supplemental_file.pdf]

SUPPLEMENTARY DATA

DIONYSUS: a database of protein-carbohydrate interfaces

Aria Gheeraert[1], Thomas Bailly[1], Yani Ren[1,2], Ali Hamraoui[1,3], Julie Te[1], Yann Vander Meersche[1], Gabriel Cretin[1], Ravy Leon Foun Lin[1], Jean-Christophe Gelly[1], Serge Pérez[4], Frédéric Guyon[1] and Tatiana Galochkina[1,*]

[1] Université Paris Cité and Université des Antilles and Université de la Réunion, INSERM, BIGR, F-75015 Paris, France

[2] Université Paris-Saclay, INRAE, MetaGenoPolis, 78350, Jouy-en-Josas, France

[3] Institut de biologie de l'École normale supérieure (IBENS), École normale supérieure, CNRS, INSERM, PSL Université Paris, 75005 Paris, France

[4] Centre de Recherches sur les Macromolécules Végétales, University Grenoble Alpes, CNRS,UPR 5301, Grenoble, France

* To whom correspondence should be addressed. Tel: +33(0)1 81 72 43 30 ; Email: tatiana.galochkina@u-paris.fr

Carbohydrate binding site annotation **1**
  Identification of carbohydrate-bringing compounds 1
  Carbohydrate chemical functions assignment 2
  Difficult cases in annotation of glycosylation sites 3
  Protein-carbohydrate interface quality in different specialized databases 4
DIONYSUS carbohydrate content **5**
DIONYSUS binding site content **6**
Non-sequential binding site alignment and scoring method **7**
Hierarchical spectral clustering of carbohydrate binding sites **8**
REFERENCES **9**

Carbohydrate binding site annotation

Identification of carbohydrate-bringing compounds

In its current state, PDB has three major problems in distinguishing carbohydrates from other ligand types:

1. Ligands annotated as "saccharide" in PDB do not include some nucleotide sugars such as UDP-glucose (PDB ligand code UPG, see Figure S1) which is classified as "non-polymer".
2. There is no canonical way to annotate glycoconjugates containing several carbohydrate residues. For example, modified acarbose hexasaccharide has five sugar cycles in its structure

under the single PDB ligand code ABC. Such heterogeneity in ligand naming hinders analysis and comparison of sugars and their binding sites.

3. A single polysaccharide is sometimes broken down into different chains and entities (e.g. structure 5TPC)

We have used two approaches to address these problems. First, we have identified a "core" set of saccharide residue names correctly annotated in the PDB and cross-references in various databases. Then, we have semi-manually parsed all the chemical compounds of the PDB in order to identify carbohydrate-containing ligands.

The first "core" set consists of 168 carbohydrate components, where both the _pdbx_chem_comp_identifier and the _pdbx_chem_com_feature section provide detailed carbohydrate-specific information. This includes common names, SNFG carbohydrate symbols assigned by the GLYCAM Molecular Modelling Library (https://github.com/glycam-web/gmml), IUPAC carbohydrate symbols attributed by pdb-care(1), as well as information about carbohydrate isomer, ring structure (furanose or pyranose), anomeric configuration, and primary carbonyl group (aldose or ketose).

The second "exhaustive" dataset was obtained through the analysis of each chemical component cataloged within the wwPDB Chemical Component Dictionary. A compound is considered as sugar-like if: (i) molecular graph has cycles of four carbons and one oxygen (potentially furanose) or five carbons and one oxygen (potentially pyranose) and (ii) at least one atom of the cycle brings one or more of the following groups: -OH, -OR, -CH2OH or -CH2OR. Each carbohydrate cycle was then considered as a separate moiety for the binding site analysis.

Our carbohydrate definition includes a broad range of compounds, notably all nucleosides and their derivatives. Nevertheless, 67 components categorized as saccharides by the PDB do not align with our criteria and were eliminated from consideration. These components fall into two categories: acyclic saccharides (e.g., 2FP) and sulfur or nitrogen saccharide derivatives (e.g., 0YT). To maintain data consistency, we deliberately choose to exclude these saccharides from our analysis and to focus on cyclic saccharides containing an oxygen atom within their cycle. In our partial copy of ProCarbDB, we discovered seven components that were not incorporated into our analysis. Six of these are acyclic saccharides, while one, residue OPG, does not fit our criteria due to its heptose structure. Given its unusual geometry, we opted to disregard it in our analysis. We have also explicitly excluded RNA and DNA-forming polymer components from this study, since the formation of such interactions differs from protein-carbohydrate recognition.

Carbohydrate chemical functions assignment

Many carbohydrates carry additional functional groups that can influence their chemical properties and interactions with proteins. Therefore, we annotated carbohydrate moieties with respect to the presence of specific atoms such as halogen (PDB ID 00A), sulfur (PDB ID 01A) or selenium (PDB ID 0U1); groups within the carbohydrate structure (phosphate (PDB ID 00A), sulfate (PDB ID 3LJ), vanadate (PDB ID AD9), amide (PDB ID 0AI), amino acid (PDB ID 0UM), lipid (PDB ID 03F)) or bond/charge annotations (such as charged (PDB ID 104) or aromatic (PDB ID 07Y)).

Finally, we classified nucleobase-containing residues using a simplified annotation. We annotated a carbohydrate moiety as part of a nucleoside if one of its substituents is a pyrimidine ring. If, in addition, the residue contains a phosphate moiety, we also annotate it as a nucleotide.

Difficult cases in annotation of glycosylation sites

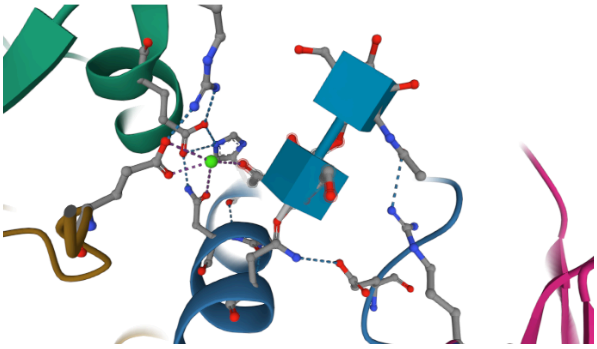

Figure S1. Resolution error in protein structure 6TVF. Carbohydrate residue NAG1 (chain S) is covalently bound to residue N82 of protein chain H forming a C-O bond instead of the expected C-N bond.

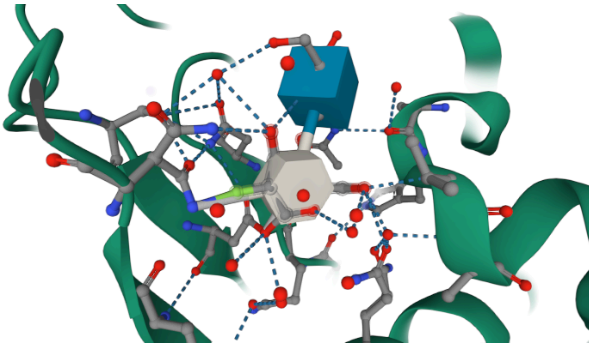

Figure S2. Example of a carbohydrate reaction intermediate for hen egg white lysozyme (PDB ID: 1H6M).

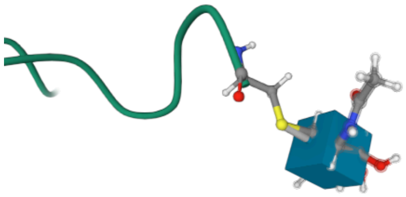

Figure S3. Example of S-glycosylation (PDB ID: 2KUY).

Finally, we use the same nomenclature for carbohydrates covalently linked to peptidic aglycons and glycosylations. The only difference arises when assigning glycosylation types, since peptidic aglycons generally comprise non-standard residues. Such cases are handled appropriately (see example 3VFK).

Carbohydrates covalently linked to non-peptidic aglycons are typically assigned a specific ligand ID in the PDB. In these cases, if the aglycon is covalently attached to the protein, the entire ligand is considered a “glycosylation head” and its glycosylation type is classified as “other.” Although we could not retrieve an exact example of this phenomenon, Coenzyme A is successfully assigned to a glycosylation head in structure 1CQI.

Additionally, we can identify rare cases where sugar-bringing molecules are covalently attached to non-sugar-bringing molecules, which are then covalently attached to a protein. An example is protein 4IZ6, where residue S575 is linked to 4'-phosphopantetheine (ligand ID: PNS), which is then attached to 5'-deoxy-5'-({[2-(2,3-dihydroxyphenyl)ethyl]sulfonyl}amino)adenosine (ligand ID: 1HZ). In the current version, this is annotated as a “glycosylation body” with the glycosylation type also classified as “other.”

Protein-carbohydrate interface quality in different specialized databases

Table S1. Number and proportion of protein-carbohydrate complexes from different specialized databases (as of January 2024) containing protein-carbohydrate interfaces of high resolution quality belonging to “Refined dataset” as defined in Materials & Methods.

	High quality	Total	% of high quality structures
All structures	31437	46984	67
CAZy	3783	4932	77
Unilectin3D	1494	1763	85
SAbDab	259	1025	25

DIONYSUS carbohydrate content

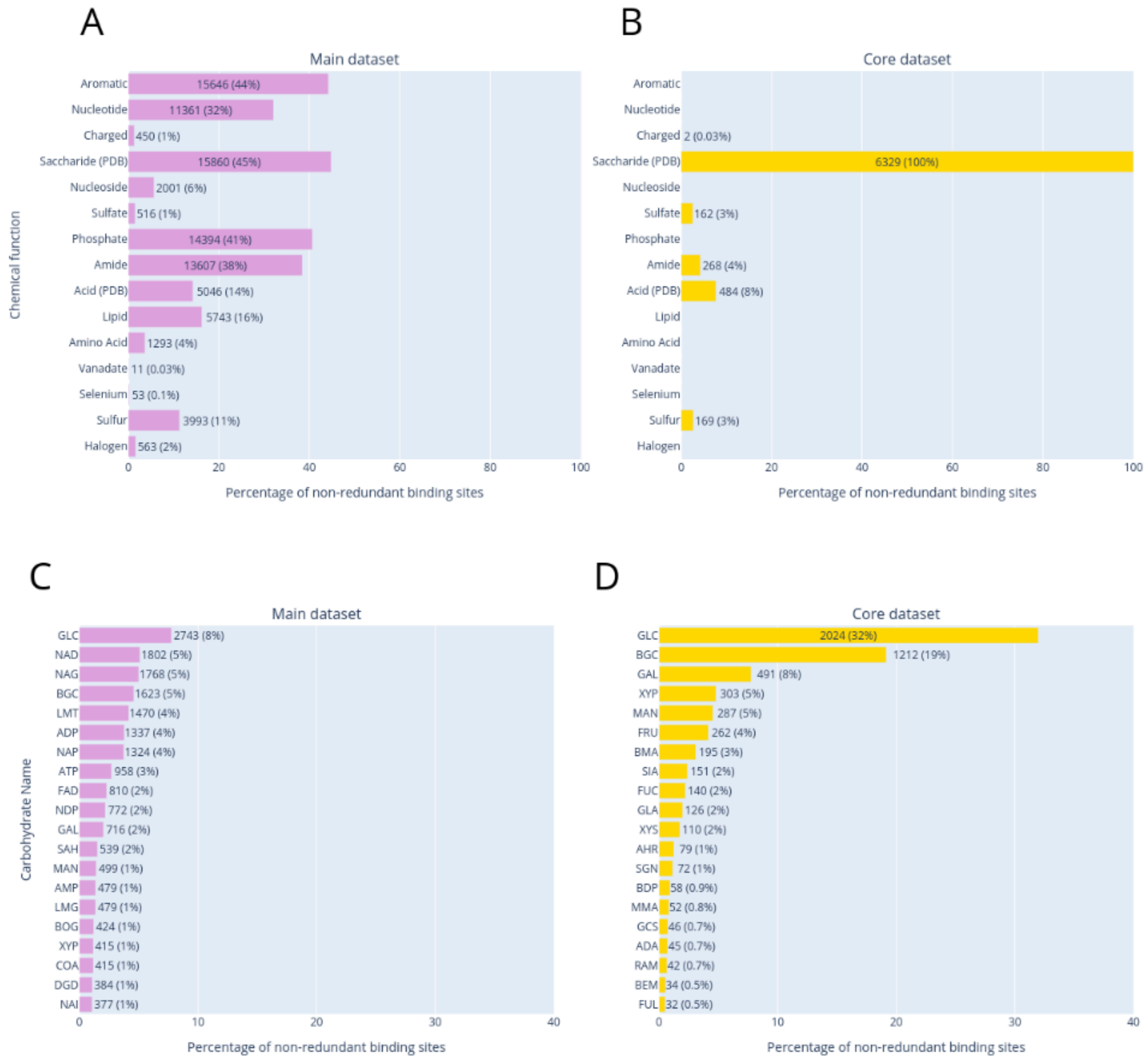

Figure S4. Distribution of chemical functions in (**A**) the non-redundant main dataset (i.e. all components except polynucleotides) (**B**) the non-redundant core dataset with only the highest quality sites. (**C** and **D**) Bar plot of the twenty most common carbohydrate monomers in each dataset. Numbers correspond to January 2024.

In Fig. S4 we report distribution of various chemical functions found in sugar components after redundancy elimination. Despite removing DNA and RNA from our dataset, the most common carbohydrate functions correspond to nucleobases: 32% nucleotides and 4% nucleosides. This is easily explained by the fact that nucleotides such as AMP/ADP/ATP or NADH/NADH are ubiquitous in biological pathways and have been extensively studied. As a result, only 45% of the binding sites we identified are formed by components labeled as saccharide by the PDB. Furthermore, the four most common chemical functions are all related to nucleotides, aromatic (nucleobases, 43%), phosphate (41%), amide (each nucleobase except adenine 39%) and acid (15%, which must be underestimated in PDB annotations). Liposaccharides represent 13% and often correspond to crystallographic adjuvants

such as dodecyl-β-D-maltoside or digalactosyldiacylglycerol. Sugars containing sulfur represents 10% of the dataset with the most common being nucleotides/nucleosides with a sulfur substituent such as S-Adenosyl-L-homocysteine or Coenzyme A while sulfates represent only 1% of the dataset.

Among high-quality sugar binding sites formed by one of 168 carbohydrate residues from the “core” list, we detect 19,816 different binding sites. The most common chemical function is amide (31%), which is explained by the presence of N-acetylated sugars in the dataset. The second most common chemical function is acid (5%) and is explained by the presence of sialic acid and sulfates. In the core dataset, by contrast to the main dataset, most sulfur-containing sugars are sulfates, the most common being 6-O-sulfo-N-sulfo-α-D-glucosamine. Only two binding sites contain a charged sugar and correspond to two deprotonated sulfate sites.

DIONYSUS binding site content

After redundancy elimination, we identify 6,723 different binding sites falling into one of the categories: lectin, CAZy, CBM, antibody and others (Fig. S4).

Among all carbohydrate binding sites, 13.5% do not have full occupancy, 7.3% miss carbohydrate atoms and 0.14% miss ring carbohydrate atoms, 9.0% contain close contacts and 0.11% clashes. 8.3% contains potential artifacts and 5.0% contains potential artifacts in a structure with 10 or more of the same compound.

Among all the resolved interfaces almost 70% have at least one identical binding site in the PDB in terms of sequence identity, ligand name and structural similarity score. This holds true for 40.4% of antibody sites, 60.4% of CBM sites, 63.5% of other sites, 65.3% of CAZys sites and for 77.7% of lectin carbohydrate binding sites.

While the diversity of carbohydrate binding sites in lectins, CBMs and CAZys is quite similar (respectively 886, 810 and 784 different CBS), we detect only 31 different antibody-carbohydrate interfaces after filtering. This is easily explained by three factors: i) antibodies are less studied than enzymes and lectins, ii) antibody CBS typically involve a glycosylated portion of another protein; those sites are discarded in the current analysis and iii) antibody often target carbohydrates in composition of more complex molecules, which do not make part of our “core” dataset (e.g., 3-Deoxy-d-manno-oct-2-ulosonic acid (KDO) on the surface of Gram-negative bacteria).

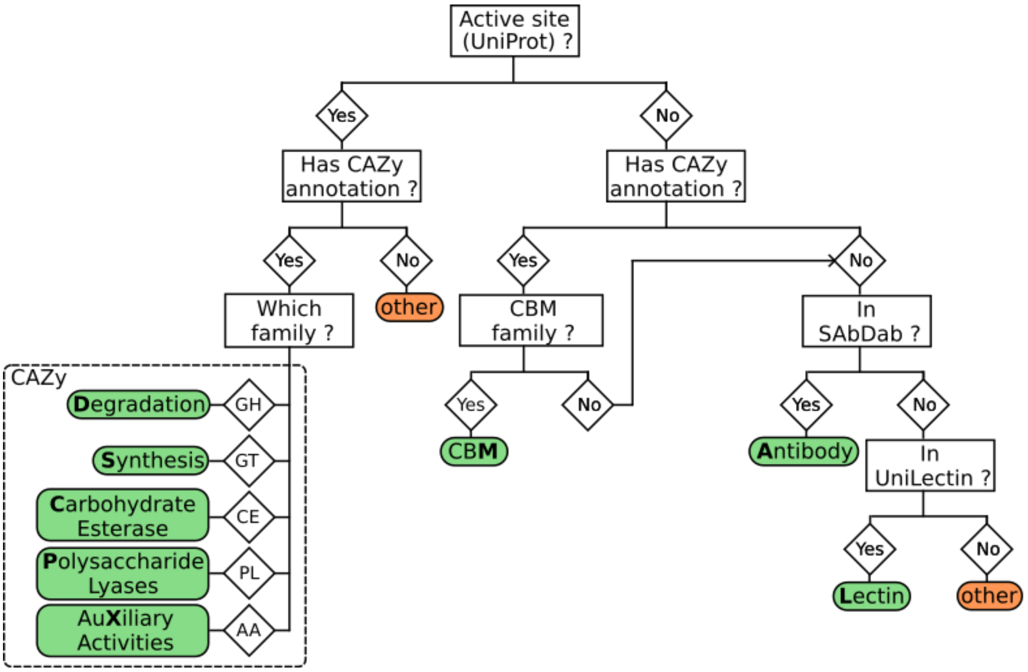

Figure S5. Site categorization according to external annotations.

Non-sequential binding site alignment and scoring method

First, for each CBS (as defined above), we assign atom types according to their physico-chemical properties. Protein atom types are determined based on the residue and atom names and include C_α, C_β, backbone oxygen, backbone nitrogen, aromatic side chain carbon, non-aromatic side chain carbon, side chain oxygen, and side chain nitrogen. For the carbohydrate ring atoms we attribute three different types: carbohydrate ring oxygen, C1 or other carbohydrate ring carbon. To ensure robustness of the subsequent calculations, if a surface protein atom is located closer than 7 Å from the carbohydrate ring, we take the C_α atom of the corresponding residue into consideration. Then, we perform a comparison of two binding sites S_1 and S_2 by computing an optimal mapping for the atoms of the same type (assigned as described above) with minimal possible distortion of the interatomic distances. Let us denote by M one-to-one mapping between atoms of S_1 and S_2 consisting of a set of atom pairs (i_1, i_2), where $i_1 \in S_1$ and $i_2 \in S_2$. The mean distortion is then calculated as:

$$MD(S_1, S_2) = \frac{1}{|M|^2} \sum_{i,j} |dist(i_1, j_1) - dist(i_2, j_2)|$$

where $dist(i_1, j_1)$ represents the Euclidean distance between atoms i_1 and j_1.

The comparison algorithm searches for the largest mapping such that the mean distortion between structures is below a given threshold noted Δ_{max}. It solves the following optimization problem:

$max_M|M|$ such that $MD(S_1, S_2) \leq \Delta_{max}$

using the maximum clique approach applied to the correspondence graph. Nodes of the correspondence graph are pairs $i = (i_{1,} i_2)$ and an edge connects node i to node j if $i_1 \neq i_2$ and $j_1 \neq j_2$ and the pairwise distortion is less than 1 Å. The details of the underlying algorithm are described in (28).

We set the precision of this algorithm to 1 Å and initiated the minimal clique using a minimum of four carbohydrate atoms.

In the present study, we use two metrics to assess the similarity between two binding sites: the coverage and the score. The alignment length is defined as the number of atoms in the maximum correspondence graph. Coverage, which serves as a similarity measure, is calculated as the alignment length divided by the size of the smaller binding site. The 'score' is then derived by weighting individual components of the coverage with Δ_{max}-$|dist(i_1, j_1) - dist(i_2, j_2)|$

Hierarchical spectral clustering of carbohydrate binding sites

For each similarity matrix, we calculate the leading n eigenvalues from the normalized Laplacian of the graph at each clustering interval, where n=max(20,Nelements). The ideal number of clusters for this partitioning is ascertained by optimizing the relative eigengap57. At each step, we use this optimal number of clusters to perform spectral clustering. The final clustering phase in the spectral embedded space uses k-means, conducted over 10 iterations. This method also enables the identification of each cluster representative, by identifying the closest point to the centroids deduced from the k-means algorithm. Should a representative fall outside the cluster or the cluster comprises only a single binding site, it is designated as an outlier. For each cluster not considered an outlier, we compute the mean score; clusters achieving a mean score above 0.55 are considered of sufficient quality, while others are re-clustered iteratively. When a cluster is considered of sufficient quality, we control that each element has at least an alignment score of 0.5 with respect to the representative binding site. If a site does not match this criterion, we exclude it from the cluster and consider it an outlier. When all sites are either clustered or outliers, all existing outliers are merged into a new group, and the hierarchical clustering is repeated for this group. This process is performed again while new clusters of sufficient quality emerge. This procedure is summarized in Figure S6.

Outlier CBS are then aligned against all representative CBS to show optimal alignments. Then we select clusters considering the union of the top three clusters based on alignment scores with the representative CBS, with any cluster that achieves an alignment score exceeding 0.65. Following this selection, each binding site is compared to every site within these identified clusters.

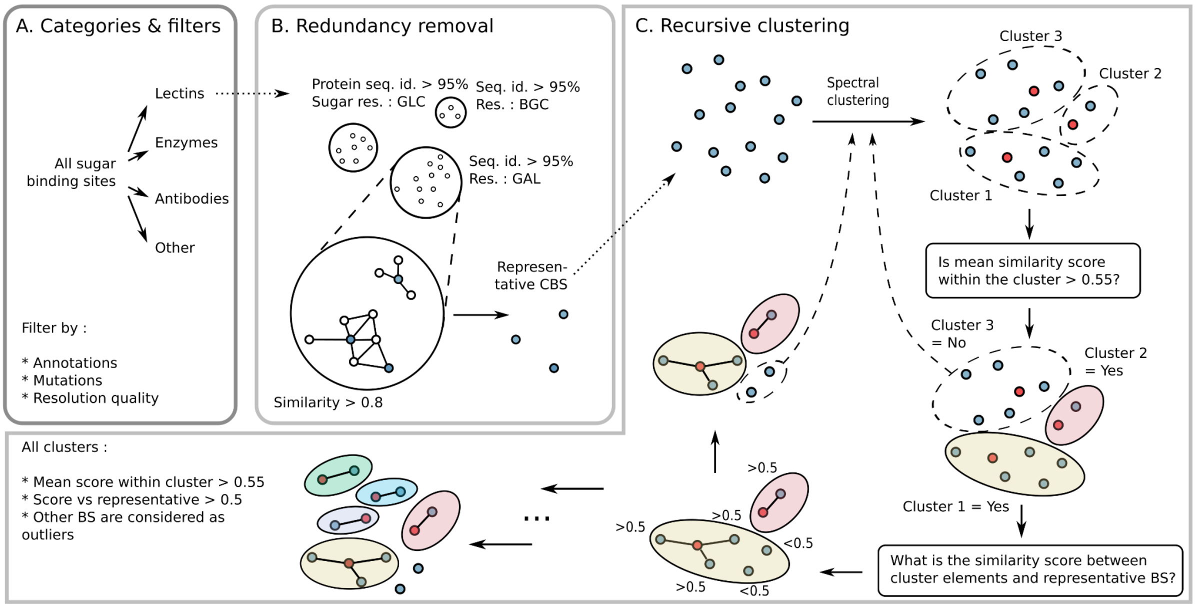

Figure S6. Summary of the hierarchical spectral clustering procedure

Table S2. Number of clusters, clustered sites and outliers in each site class.

Category	Degradation	CBM	Lectin	Polysaccharide Lyase	Antibody	Synthesis	Auxiliary Activities	Carbohydrate Esterase
Number of Clusters	68	91	84	7	4	3	1	0
Clustered Sites	256	267	435	15	8	6	2	0
Outliers	304	502	429	27	23	8	2	7

Finally, we performed clustering quality assessment using ratio between two measures: the inside score and the outside score as defined below:

- Inside score: Average score within the cluster
- Outside score: Average score between elements in the cluster and elements outside the cluster

Then, cluster quality is assessed based on the following thresholds:

- Above 2: High
- Between 1.5 and 2: Good
- Between 1.1 and 1.5: Medium
- Below 1.1: Low

REFERENCES

1. Lütteke,T. and von der Lieth,C.-W. (2004) pdb-care (PDB CArbohydrate REsidue check): a program to support annotation of complex carbohydrate structures in PDB files. *BMC Bioinformatics*, **5**, 69.
